# Insights into Candida auris dispersal cells and their impact on antifungal resistance

**DOI:** 10.1186/s12866-025-04055-8

**Published:** 2025-05-29

**Authors:** Bahgat Fayed

**Affiliations:** Department of Chemistry of Natural and Microbial Products, Pharmaceutical and Drug Industries Research Institute, National Research Centre, 33 El Bohouth Street, P.O. 12622, Dokki, Giza, Egypt

**Keywords:** *Candida auris*- resistance, Caspofungin, Biofilm

## Abstract

The emerging *Candidozyma auris* (formerly known as *Candida auris*, *C. auris*) has caused several outbreaks globally. While several studies explored the resistant biofilm formed by *C. auris*, little is known regarding the cells dispersed following biofilm formation. Herein, I investigated and characterized the cells dispersed from *C. auris* biofilms. Cells dispersed from biofilm developed in 96 well plate were isolated and counted. The antifungal susceptibility testing showed that the dispersed cells display similar antifungal susceptibility as the parent planktonic cells, except amphotericin B. Gene expression analysis performed by quantitative real-time PCR indicated that dispersed cells can express genes coded for antifungal resistance (*ERG2*,* ERG6*,* ERG11*,* FKS1*,* CHS1*,* CHS2*,* CDR1*,* MDR1*) more than the parent planktonic cells. It was observed that dispersed cells can acquire resistance to caspofungin faster than the parent planktonic cells once exposed to caspofungin at sub MIC level. Furthermore, biofilms formed by dispersed cells displayed significantly higher metabolic activity, as indicated by the XTT analysis. To provide more insight, I explored the expression of genes coding for biofilm initiation and maturation and the data obtained indicated that dispersed cells overexpressed *ALS5* and *KRE6* genes. Further, GC-MS analysis indicated that dispersed cells exhibit altered metabolic profile that enhance cells survivability under stress and nutrient limit condition. The presented study is the first to explore *C. auris* dispersed cells and indicated that they are not able to revert to the planktonic mode once released from the biofilm.

## Introduction

Globally, multidrug-resistant *Candida* species are rapidly spreading, which threatens and hampers the efficacy of antifungal drugs [1]. The pathogen *C. auris* has emerged in recent years, causing outbreaks in long-term health care facilities and acute care settings, with mortality rates exceeding 60% for immunocompromised patients [2–4]. While azoles are considered the front-line treatment for systemic infections caused by most *Candida* species, echinocandins are the first-line drug against systematic *C. auris* infections due to the high resistance profile of *C. auris* to azoles [2, 5]. Furthermore, 41% of *C. auris* clinical isolates were found to be multidrug resistant, while approximately 4% were pandrug resistant [6].

One of the major virulence factors of *C. auris* is its ability to form biofilms on medical devices and hospital surfaces, which not only enhances its survival but also contributes to its resistance to antifungal treatments [5]. Biofilm-associated cells have been shown to resist antifungal drugs at concentrations up to 1,000 times higher than those needed for planktonic cells, further complicating infection control [7]. Additionally, the dispersion of cells from yeast biofilms is another milestone in the biofilm life-style. Biofilm development includes the adherence phase, followed by proliferation, maturation and dispersal phases [8]. In *C. auris*, planktonic yeast cells initially adhere to surfaces and proliferate to form dense aggregates. These cells then develop into a structured biofilm primarily composed of yeast-form cells. At later stages, *C. auris* biofilms can release dispersed yeast cells into the surrounding environment, enabling colonization of new niches and continuation of the biofilm lifecycle [9]. The dispersion of cells from biofilms is a crucial process. For instance, cells dispersed from biofilm urinary catheters will have direct access to the bloodstream and cause severe candidaemia [10]. Furthermore, Uppuluri et al. demonstrated the ability of dispersed yeast cells to adhere and induce damage to endothelial cells better than planktonic cells. Additionally, dispersed yeast cells exhibit a greater resistance profile, enhanced virulence and greater ability to form denser biofilms than planktonic cells [11]. Finally, transcriptome sequencing analysis of *C. albicans*-dispersed cells demonstrated that they have distinct expression patterns compared to both biofilms and planktonic cells, which provides them with the ability to invade and survive within new sites in the host and even under nutrient starvation conditions [10]. Additionally, numerous studies have investigated the dispersion of cells from *C. albicans* biofilms and the molecular mechanisms controlling this process, while little attention has been given to the events associated with the dispersion of cells from *C. auris* biofilms. To address this issue, the present study explored and characterized the cells dispersed from *C. auris* biofilms. I hypothesize that cells dispersed from *C. auris* biofilms may exhibit distinct antifungal resistance and biofilm-forming capabilities compared to planktonic cells. This study aims to characterize these dispersed cells and provide insight into their role in resistance development and biofilm formation, thereby contributing to a better understanding of the mechanisms underlying *C. auris* pathogenicity.

Overall, this study provides novel and crucial insight into *C. auris* dispersed cells to expand our understanding of how to control these deadly infections that are currently spreading at alarming rates.

## Materials and methods

### Materials

Sabouraud dextrose (SD) media was obtained from HIMEDIA (Mumbai, India), RPMI-1640, PBS, caspofungin, flucytosine, amphotericin B, menadione, glass beads were obtained from Sigma Aldrich Co. (St. Louis, MI, USA). Fluconazole was purchased from Merck Millipore (Burlington, MA, USA). TRIzol™, Turbo-DNaseI were obtained from Invitrogen (Massachusetts, USA). Retroscript First-Strand Synthesis Kit, and Ribopure™ RNA were obtained from Ambion (Massachusetts, USA).

### Strain and culture

*C. auris* was obtained from the CDC, Atlanta, GA, USA (CDC_B11220). The strain was routinely maintained in SD media and incubated at 37 °C. For storage at − 80 °C, 30% glycerol stocks were used for storage.

### Isolation of cells dispersed from *C. auris* biofilm

*C. auris* biofilms were developed by loading 1 × 10^6^ cells/ml in RPMI-1640 buffered with 0.165 M MOPS supplemented with 2% glucose and then incubating for 24 h at 37 °C in 96-well flat-bottomed microplates [12]. Following incubation, the supernatant containing floating cells was removed, the formed biofilm was washed 3 times with PBS, fresh media was added, and the biofilm was incubated for an additional 24 h under the same conditions. The next day, the cells dispersed from the biofilm in the upper supernatant were collected, counted by a hemocytometer and maintained in SD media or stored in 30% glycerol for further use. The experiment was conducted in triplicate to ensure reproducibility.

### Antifungal susceptibility testing

Antifungal susceptibility testing was performed for both the *C. auris* dispersed cells and the parent planktonic cells by measuring the minimum inhibition concentration (MIC_50_) following the modified Clinical and Laboratory Standards Institute (CLSI) as previously described by Fayed et al., 2020 [2]. Briefly, *C. auris* strains at 10^4^ CFU/ml obtained from overnight culture at 37 °C were incubated with 100 µl of the tested antifungal drugs (fluconazole, caspofungin, amphotericin B, and flucytosine) in a flat-bottom 96-well plate (Costar, USA). The concentrations used for fluconazole, caspofungin, amphotericin B, and flucytosine were (25, 12.5, 6.25, 3.12, 1.56 µg/ml), (100, 50, 25, 12.5, 6.25 ng/ml), (10, 5, 2.5, 1.25, 0.612 µg/ml), and (20, 10, 5, 2.5, 1.25 µg/ml) respectively. The mixture was incubated for 48 h at 37 °C without shaking. A microplate reader was used to evaluate *Candida* growth by measuring the turbidity at OD_600_. The obtained MIC_50_ value was defined as the lowest concentration at which the microbial growth decreased by 50% compared to that of the control. The experiment was performed in triplicate, and the average value was obtained.

### Assessment of resistance to Caspofungin acquired by dispersed *C. auris* cells

*C. auris* dispersed and planktonic cells (1 × 10^6^ cells/ml) were allowed to grow in 1 ml SD broth for 48 h at 37 °C in the presence of 25 ng/ml caspofungin [2]. This concentration is intended to affect the resistance mechanisms without necessarily killing all the cells. Following incubation, the cells were obtained by centrifugation and washed with sterile PBS, after which the caspofungin MIC_50_ was determined as previously described.

### Estimation of *C. auris* biofilm viability by the XTT method

*C. auris* cells, either planktonic or dispersed, were adjusted to 1 × 10^6^ cells/ml and then incubated in 100 µl RPMI-1640 at 37 °C for 2 h in 96-well microplates. The supernatant was removed, and the attached cells were washed with PBS. Then, fresh media was added, and the cells were allowed to incubate for an additional 48 h. Biofilms that formed at the bottom of the wells were washed with sterile PBS two times. One hundred microliter of 5 mg/mL XTT (2,3-(2-methoxy-4-nitro-5-sulphophenyl)-5-[(phenylamino) carbonyl]-2 H-tetrazolium hydroxide) and 1 mM menadione were added to the mixture in the dark and then incubated at 37 °C with shaking at 250 rpm for 30 min. The absorbance at 490 nm was recorded by a microplate reader [13]. The experiments were performed in triplicate, and the average value was calculated.

### Quantitative real-time PCR (qRT‒PCR)

The expression levels of genes coding for antifungal resistance, biofilm adhesion and maturation were quantified using qRT-PCR in two separate experiments. In the first experiment, RNA was isolated from both the parent planktonic and dispersed cells to quantify the expression of antifungal resistance genes. In the second experiment, both parent planktonic and dispersed cells were used to form biofilms. Once the biofilms were developed, RNA was extracted to analyze the expression of genes related to adhesion and biofilm maturation.

For both experiments, the cells or the biofilm were pelleted by centrifugation at 4500 RPM for 10 min, washed with PBS, and then ground in 0.5 ml TRIzol^™^ using glass beads at 4 °C for 10 min [2]. Chloroform (100 µl) was added to the ground cells, and the upper aqueous layer was obtained by centrifugation at 8000 RPM for 20 min and transferred to a new tube for RNA extraction. RNA extraction was performed using a Ribopure™ RNA purification kit according to the manufacturer’s instructions. Genomic DNA was removed during the extraction process by treating the samples with 4 µL of Turbo-DNaseI at room temperature for 25 min, after which DNase inactivation reagent was used to remove DNase. A Retroscript First-Strand Synthesis Kit (Ambion) was used to construct cDNA, and gene expression was quantified by SYBR Green PCR Master Mix (Applied Biosystems, Egypt) using an ABI Prism 7000 Sequence Detection System (Applied Biosystems). Gene expression was quantified using the 2^(−ΔΔCT)^ method [14]. Actin gene (*ACT 1*) was used as housekeeping gene. All samples were analyzed in triplicate, and the primers used in the study are listed in Table 1.

**Table 1:** List of primers used in the study

Gene name	Gene symbol	Primer sequence (5′–3′)
Actin gene	*ACT1*	FW- CGCTGGTTTCTCGTTACCAC RV- CAGCAGTGGTAGAGAAGGTGT
Lanosterol 14-alpha-demethylase synthase gene	*ERG11*	FW-CAAGTCGTTGATGGGTGATG RV- GAACGATGTCACCGGTCTTT
Gene encode C-8 sterol isomerase	*ERG2*	FW-GAGAGGCCAAGTGAAGCAGT RV-ACACAAAGCCGAATGGCAAC
Gene encode the sterol methyltransferase	*ERG6*	FW-GACGTTGGGCGACTACTTCA RV- GCAAGCCCGATCTTCTCCAT
Agglutinin-like sequence proteingene *ALS5*	*ALS5*	FW- CCTTCTGGATCGGACACAGT RV- AGTTGTGGTGGAGGAACCAG
1,3-beta-D-glucan synthase gene	*FKS1*	FW- CGAAGAACACGGTCAGGACA RV- CCTCAGGGGTCAAGACGTTC
Chitin synthase I gene	*CHS1*	FW- CGCCGTTTACAACCTTTGGA RV- TGAGAAGCAACGAGTGGGTTT
Chitin synthase II gene	*CHS2*	FW- GGTGCCACGGAGTTAGACAA RV- AGTCAGCACGAGCTTTGACA
Gene encodes Beta-glucan synthesis associated protein KRE6	*KRE6*	FW- CAGAAGAGCGTTGGGAGGAG RV- GCTCCCGAGTAGAAGAGCAC
Gene encodes ABC multidrug transporter MDR1	*MDR1*	FW- TCAGACACCCCCGTTGATCT RV- TCTTCTCCCTCCCAGTCCAC
Gene encodes efflux pump gene *CDR1*	*CDR1*	FW- GCCAGGTTTCTGGATTTTCA RV- GGCCACAAGTTTGACCACTT
Gene encodes GPI-anchor protein 26	*PGA26*	FW- CACTGAGCCATGTGTTGTGC RV- GTGGACTCACCAGCAACAGT
Gene encodes GPI-anchor protein 52	*PGA52*	FW- TCCACGATTACCAAGGCACC RV- ATCGAACACAACGCCTCCAA
Gene encodes GPI-anchor protein IFF4	*IFF4*	FW- CTCAACATCAACCCTGGCGT RV- AGAAGCAGCCAATCCGTTGT
Gene encodes a mycelial surface antigen	*CSA1*	FW- TCCTCCAAGAACATCCGCAC RV- GAGGAAGTGGCTCTGGTGAC

### Metabolite extraction and chromatographic analysis using GC–MS

*C. auris* dispersed cell were allowed to grow in 250-mL Erlenmeyer flasks containing 50 mL of SD broth media for 3 days at 30 °C. Following incubation, the supernatant was separated by centrifugation for 20 min at 4000 rpm. The resulting supernatant was extracted with 100 mL ethyl acetate. After the extraction step, the organic phases were dried on anhydrous MgSO4, filtered, and concentrated under reduced pressure. The parent *C. auris* planktonic cells served as control. Chromatographic analysis using GC-MS was performed in central lab (Ain Shams University, Cairo, Egypt) with an Agilent Technologies 7890B GC System coupled to a 5977 A Mass Selective Detector. An HP-5MS capillary column (30.0 m × 0.25 mm ID × 0.25 μm film) was utilized, with helium as the carrier gas at a pressure of 8.2 psi and a 1 µL injection. Initially, 300 µL of N, O-Bis(trimethylsilyl)trifluoroacetamide was added to the sample, which was then heated in a water bath at 80 °C for two hours before injection into the GC/MS. The sample analysis began with the column held at 60 °C for 5 min post-injection. The temperature was subsequently increased to 300 °C at a rate of 20 °C per minute, followed by a 5-minute hold. The injection was performed in split mode with a split ratio of 5:1 at 300 °C. The MS scan range was 50–550 atomic mass units (AMU) under electron impact ionization (70 eV), with a solvent delay of 8.0 min. The constituents were identified by mass fragmentations using the NIST mass spectral search program for the NIST/EPA/NIH mass spectral library Version 2.2 (Jun 2014). The data obtained from GC-MS were transferred to an Excel worksheet, which was subsequently filtered and organized, including metabolite names and matching area under the peak values. The area under the peak values for all metabolites was then exported to MetaboAnalyst 6.0 software for metabolomics data analysis and interpretation.

### Statistical analysis

The MIC_50_ was calculated and graphed using GraphPad Prism (8.02, GraphPad Inc., La Jolla, CA, USA). The gene expression analysis data were analyzed and graphed using an unpaired t test with Welch correction. *p* < 0.05 was considered significant. The data are presented as the means ± SDs of 3 replicates.

## Results

### *C. auris* biofilms can disperse cells at high rates

Following the maturation of *C. auris* biofilm, I observed the dispersal of cells into the surrounding medium. Using a hemocytometer, the density of these dispersed cells was calculated to be 1.28 × 10^6^ cells per well. This demonstrates that *C. auris* biofilms can actively release cells into their environment at a high rate.

### Antifungal susceptibility of dispersed cells

To assess whether the dispersed cells retain similar antifungal susceptibility profiles compared to their planktonic counterparts, MIC_50_ values were determined using a range of antifungal agents. Both cell types exhibited similar susceptibility patterns for most drugs tested. The MIC_50_ values for fluconazole were 3.46 ± 0.2 µg/ml for dispersed cells and 3.98 ± 0.5 µg/ml for planktonic cells, while for flucytosine, the values were 1.78 ± 0.32 µg/ml and 1.89 ± 0.15 µg/ml, respectively. For caspofungin, dispersed cells exhibited an MIC_50_ of 29.51 ± 2.08 ng/ml compared to 28.25 ± 1.08 ng/ml for planktonic cells. However, for amphotericin B, the dispersed cells demonstrated a slightly elevated MIC_50_ of 1.23 ± 0.17 µg/ml compared to 0.995 ± 0.058 µg/ml for planktonic cells, indicating a potential change in susceptibility upon dispersion.

### Overexpression of antifungal resistance genes in dispersed cells

**Fig. 1:**
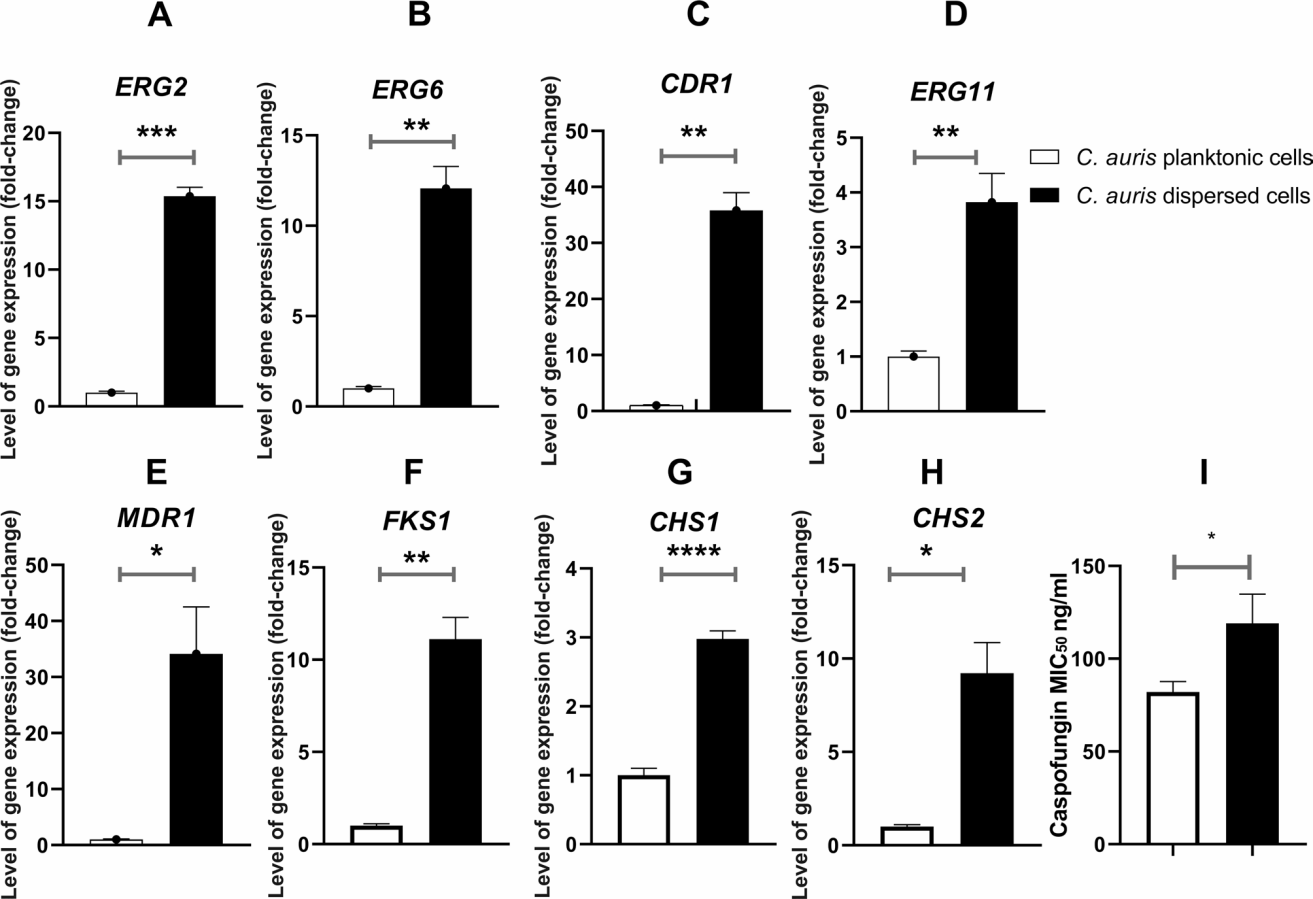
Caspofungin acquired resistance analysis (**A**) Expression analysis of *ERG2*, (**B**) *ERG6*, (**C**) *CDR1*, (**D**) *ERG11*, (**E**) *MDR1*, (**F**) *FKS1*, (**G**) *CHS1*, (**H**) *CHS2*, (**I**) Caspofungin MIC_50_. For panels (**A**)-(**H**), bars represent mean expression levels normalized to a housekeeping gene (*ACT1*). Statistical analysis was performed by an unpaired t test with Welch correction. The data display the mean ± SD of three replicas. * *P* < 0.05, ** *P* < 0.01, ***P < 0.001, represent the significant levels

To explore potential genetic differences between dispersed and planktonic cells, qPCR was performed to assess the expression of key genes associated with antifungal resistance. This analysis was conducted on dispersed cells immediately after collection, without allowing them to form biofilms. The qPCR results, presented in Fig. 1 (A-H), indicate that dispersed cells consistently overexpressed these resistance-associated genes when compared to planktonic cells. Specifically, dispersed cells overexpressed *ERG11* by 3.8 ± 0.52-fold, *ERG2* by 15.37 ± 0.64-fold, and *ERG6* by 12.07 ± 1.2-fold. For caspofungin resistance, the *FKS1*,* CHS1*, and *CHS2* genes were overexpressed by 11.11 ± 1.17, 2.97 ± 0.11, and 9.2 ± 1.63-fold, respectively. Additionally, the drug efflux pump genes *CDR1* and *MDR1* were overexpressed by 35.8 ± 3.1 and 34.15 ± 8.36-fold, respectively. This suggests that dispersed cells may possess a heightened ability to withstand antifungal treatment.

### Enhanced acquisition of Caspofungin resistance in dispersed cells

To further investigate the resistance profile of dispersed cells, I exposed both dispersed and planktonic cells to sub-inhibitory concentrations of caspofungin for 48 h. Following exposure, the MIC_50_ for caspofungin was determined for each cell type. The data revealed that dispersed cells showed tendency to acquire resistance to caspofungin more rapidly than planktonic cells, with a significant increase in MIC_50_ values after exposure. These results are shown in Fig. 1 (I), underscoring the potential for dispersed cells to develop antifungal resistance.

### Biofilm-forming capacity of dispersed cells

**Fig. 2:**
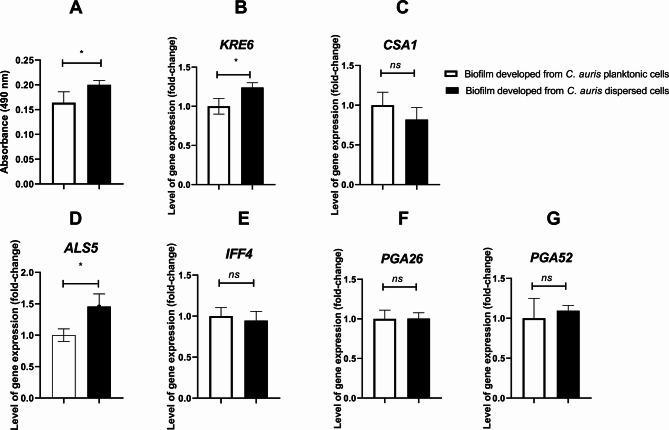
Analysis of biofilm formation. **A**) XTT analysis showing the metabolic activity of biofilm-forming cells over time., **B**) Gene expression analysis of *KRE6*, **C**) *CSA1*, **D**)*ALS5*, E)*IFF4*, F)*PGA26*, G)*PGA52.* For panels (**B**)-(**G**), bars represent mean expression levels normalized to a housekeeping gene (*ACTI*). Statistical analysis was performed by an unpaired t test with Welch correction. The data display the mean ± SD of three replicas. * *P* < 0.05, represent the significant levels

The biofilm-forming capacity of dispersed cells in comparison to planktonic cells was next evaluated. Both cell types were allowed to form biofilms under identical conditions, and biofilm viability was assessed using the XTT reduction assay, which measures metabolic activity. As shown in Fig. 2 (A), biofilms formed by dispersed cells displayed significantly higher viability, indicating that these cells are more capable of producing metabolically active biofilms compared to their planktonic counterparts.

### Expression of adhesion and biofilm growth genes

To investigate the potential differences in biofilm formation, the expression levels of adhesive and maturation genes in biofilms formed from both *C. auris* parent planktonic cells and dispersed cells were assessed. The biofilms were allowed to form under the same conditions, and RNA was extracted from these biofilms for gene expression analysis. The expression levels of genes encoding *ALS5*,* IFF4*,* PGA26*,* PGA52*, and *CSA1* were quantified as shown in Fig. 2 (B-G). The biofilms formed from dispersed cells showed a 1.46 ± 0.2-fold higher expression of the *ALS5* gene compared to those formed from parent planktonic cells. Additionally, the expression of the biofilm growth-related gene *KRE6* was 1.24 ± 0.06-fold higher in biofilms formed from dispersed cells than in those formed from parent planktonic cells, while no significant differences were observed for the other genes.

### The cells dispersed from biofilm secrete different metabolites compared to the parent planktonic cells

**Fig. 3:**
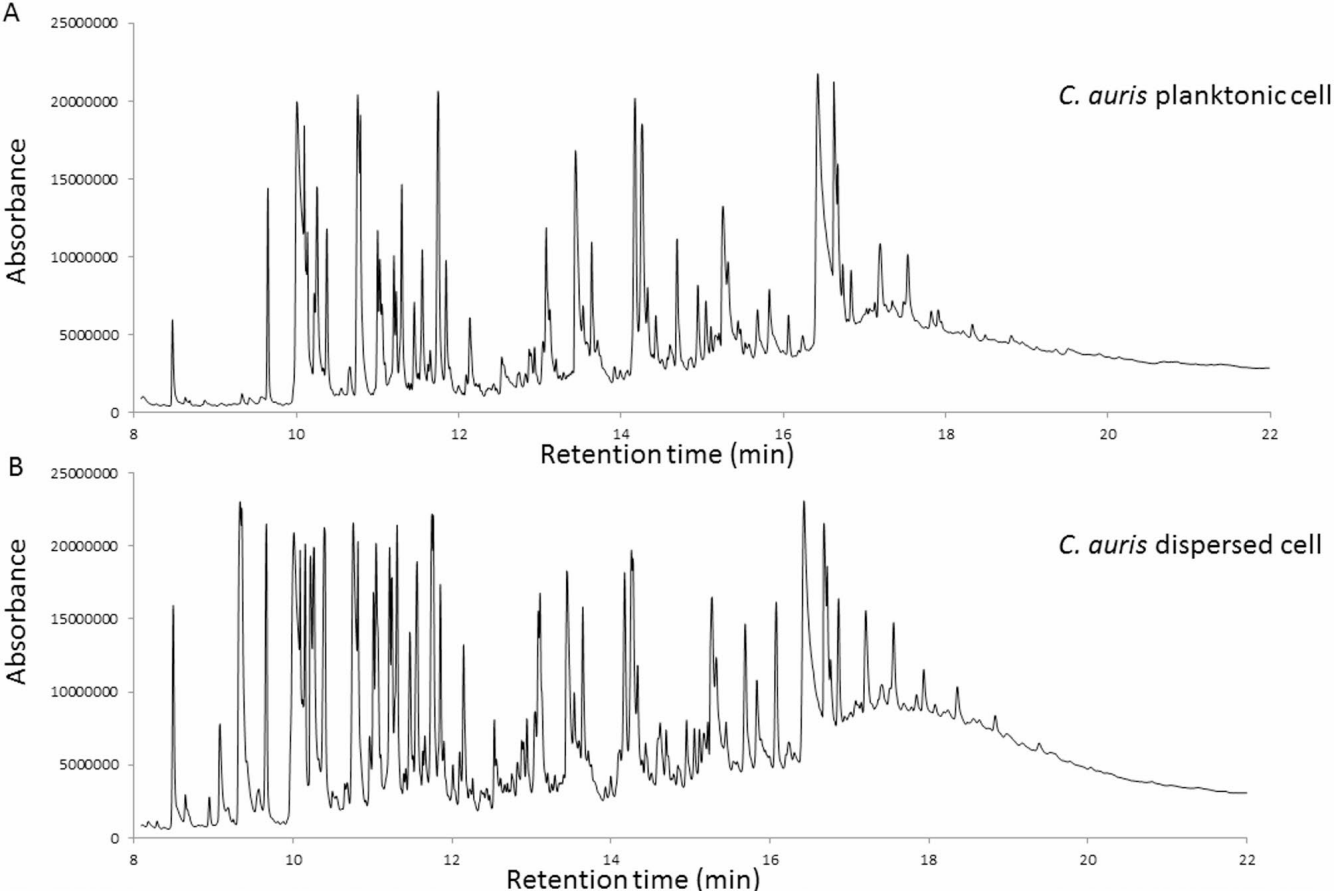
GC-MS Chromatogram showing the metabolic profile of (**A**) planktonic *C. auris*. (**B**) *C. auris* dispersed cells

**Fig. 4:**
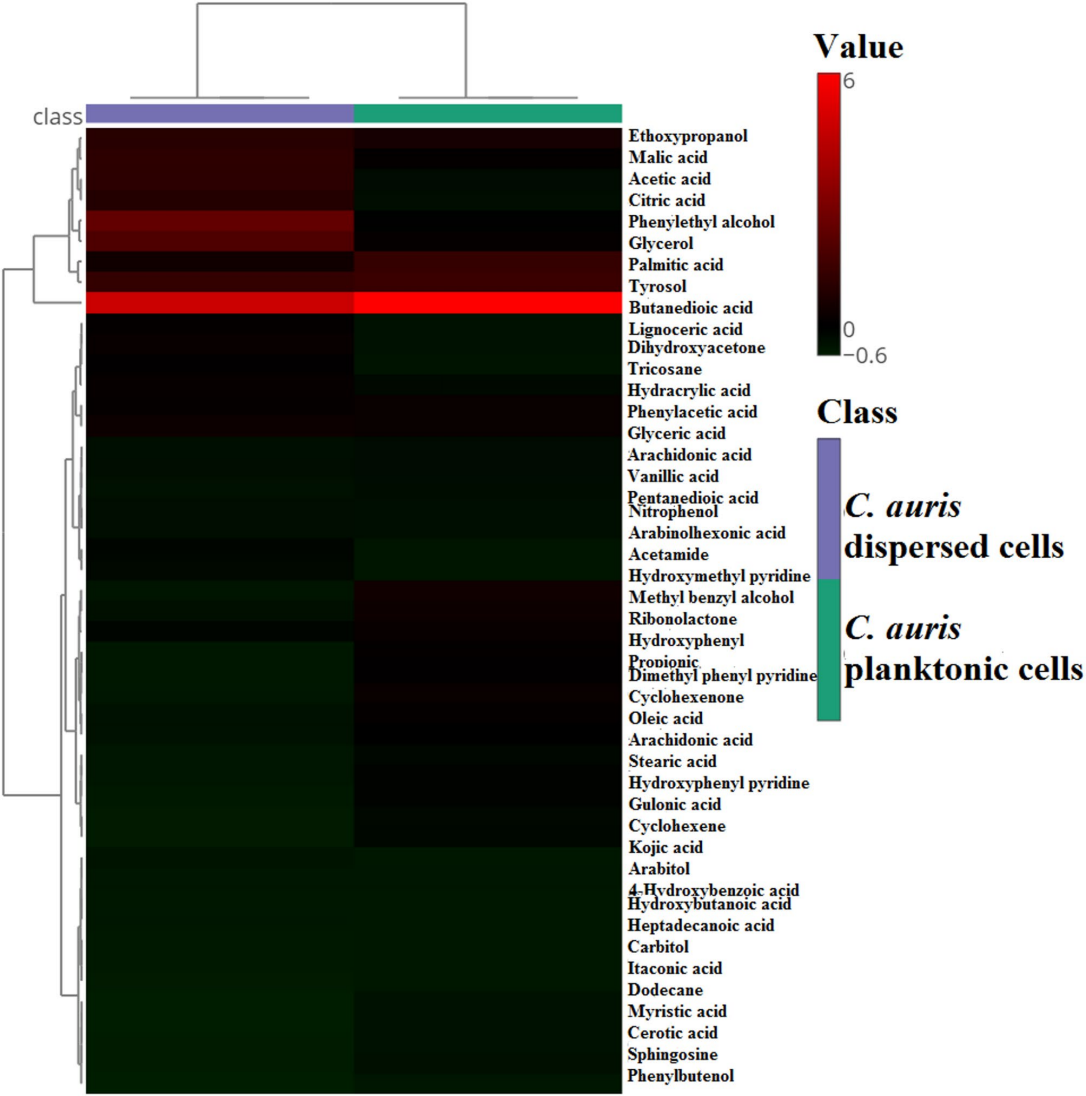
Hierarchical clustering analysis of differentially expressed metabolites from *C. auris* planktonic cells, and *C. auris* cells dispersed from biofilm. The intensity of colors indicates the expression levels of the metabolites. Each row represents a different metabolite, and each column represents a sample condition. Hierarchical clustering analysis was performed by MetaboAnalyst 6.0 software for metabolomics data analysis

**Fig. 5:**
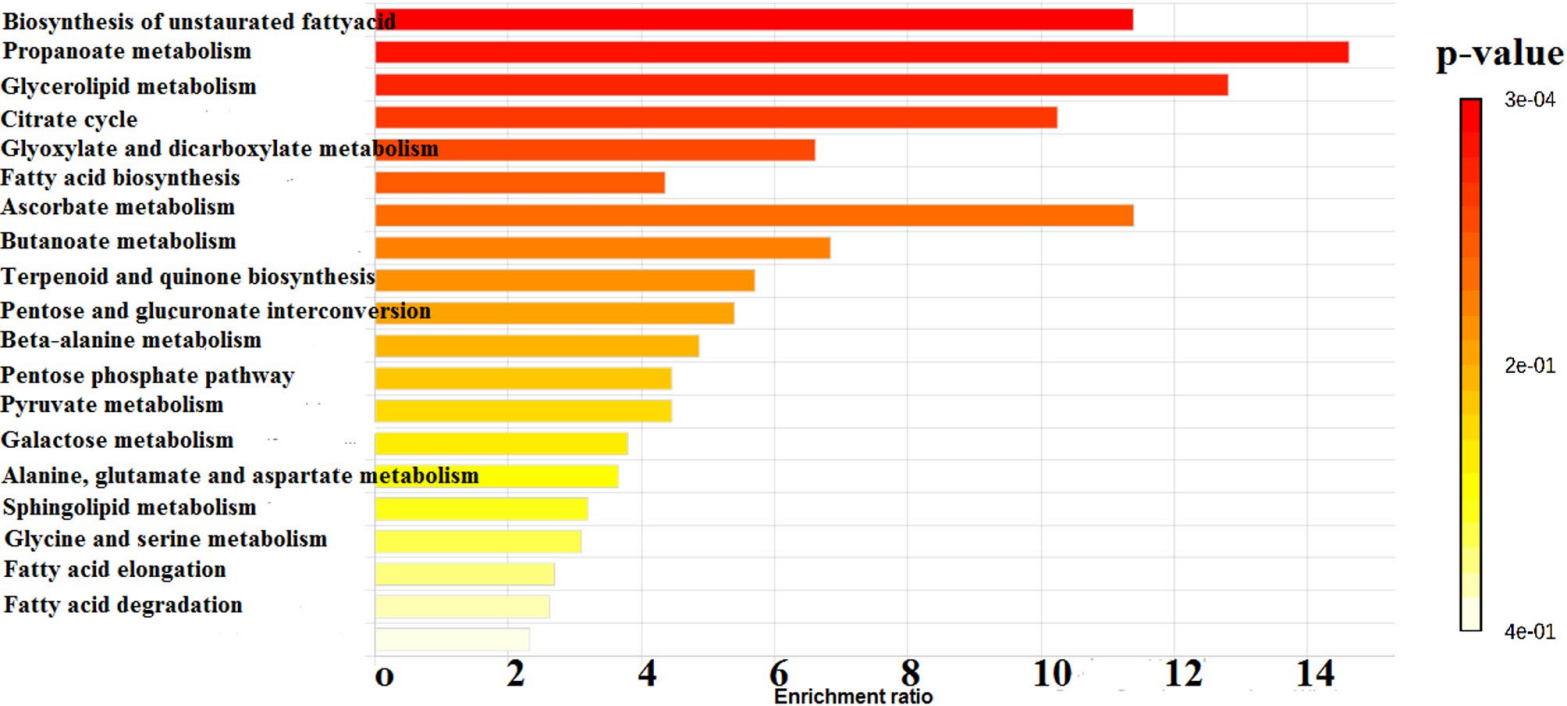
Metabolite sets enrichment ratio of *C. auris* planktonic cells. Bar graph showing enrichment ratios of various metabolite sets in planktonic cells. The intensity of colors indicates the significant enrichment. Enrichment ratios are calculated based on metabolite abundance. The graph was constructed by MetaboAnalyst 6.0 software for metabolomics data analysis

**Fig. 6:**
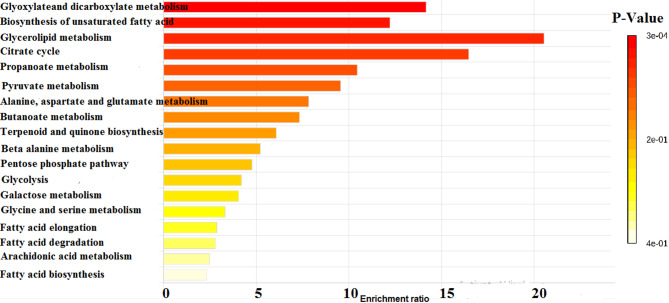
Metabolite sets enrichment ratio of *C. auris* cells dispersed from biofilm. Bar graph showing enrichment ratios of various metabolite sets in dispersed cells. The intensity of colors indicates the significant enrichment. Enrichment ratios are calculated based on metabolite abundance. The graph was constructed by MetaboAnalyst 6.0 software for metabolomics data analysis

**Fig. 7:**
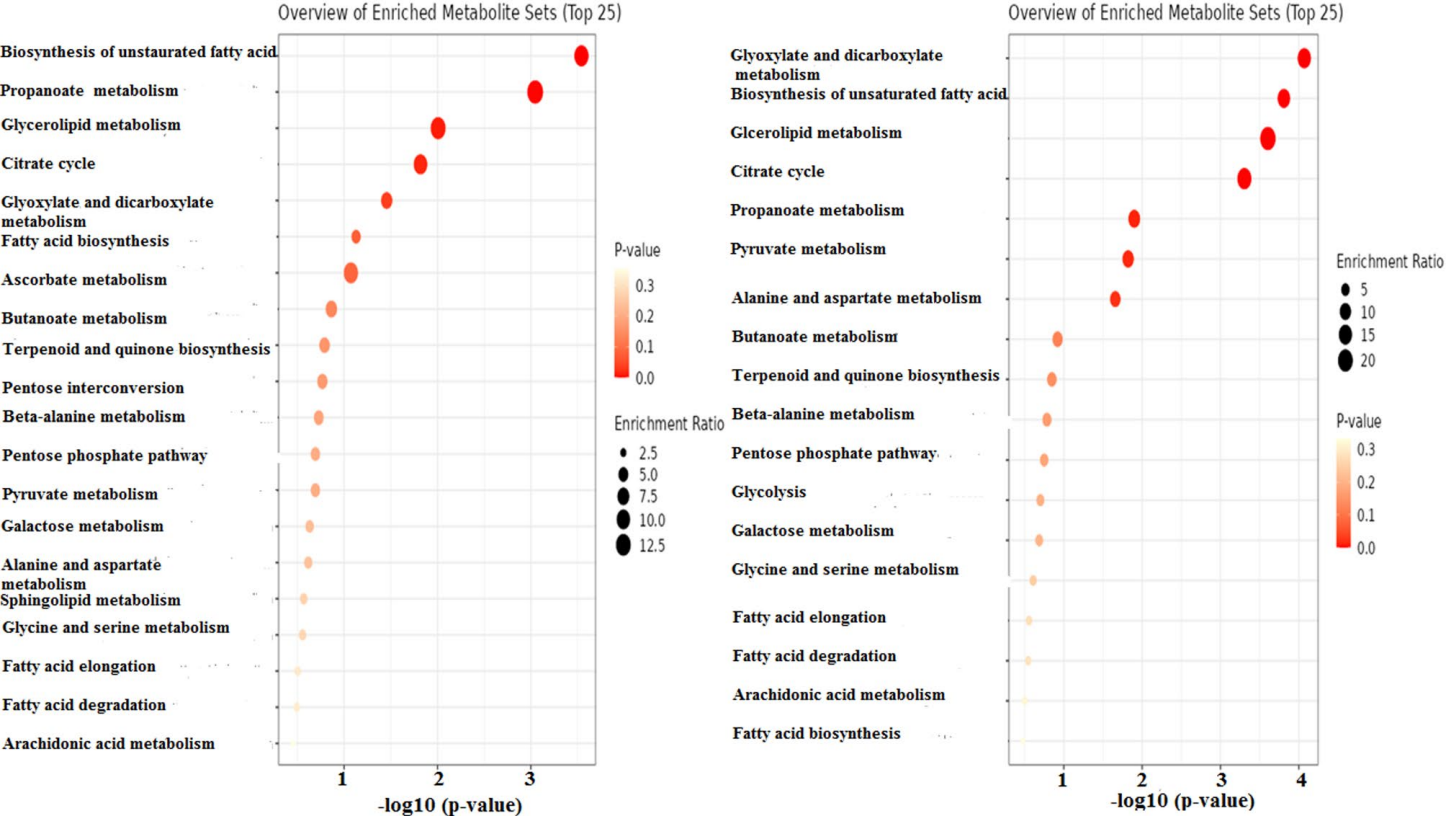
Overview of enriched metabolite sets of **A**) *C. auris* planktonic cells, **B**) *C. auris* cells dispersed from biofilm. The chart showing the distribution of enriched metabolite sets. The intensity of orange color represents the significant enrichment, while the size represents the enrichment ratio. The chart was constructed by MetaboAnalyst 6.0 software for metabolomics data analysis

To further characterize the physiological differences between dispersed and planktonic cells, metabolomic analysis using gas chromatography-mass spectrometry (GC-MS) was performed. The chromatograms, shown in Fig. 3, revealed that while both cell types secreted 22 common metabolites, the concentrations of these metabolites varied significantly. In addition, dispersed cells produced 13 unique metabolites not found in planktonic cells, as illustrated in Fig. 4; Table 2. Pathway enrichment analysis revealed that different metabolic pathways were activated in each cell type. Dispersed cells showed significant enrichment in the glyoxylate and dicarboxylate metabolism pathways, whereas planktonic cells primarily enriched fatty acid biosynthesis pathways. Moreover, glycerolipid metabolism was notably enhanced in dispersed cells, indicating their metabolic adaptation to stress and nutrient-limited environments (Figs. 5, 6 and 7). These differences in metabolic activity may contribute to the unique survival strategies of dispersed cells.

**Table 2:** Metabolites solely expressed by *C. auris* cells dispersed from biofilm

Metabolite name	Retention time (min)	Area %
4-Hydroxybutanoic acid	8.65	0.302
Phenylethyl Alcohol	9.3403	4.8358
5-Hydroxy-2-methylpyridine	9.0828	0.8189
2-Hydroxy-3-methylbutyric acid	11.4231	0.4132
Dihydroxyacetone	11.4746	1.4153
Carbitol	12.2642	0.1844
D-(+)-Arabitol	12.5331	0.3812
Ribitol	12.5618	0.2862
Heptadecanoic acid	12.4359	0.2167
Citric acid	13.1054	2.485
Itaconic acid	13.998	0.1513
Acetic acid	14.2612	2.8062
Acetamide	14.3356	0.9232

## Discussion

Microbial cells dispersed from biofilms can play a crucial role in the spread of infection in intensive care units from medical devices to human hosts and even in the spread of infection within a host [15]; however, little attention has been given to the role of cells dispersed from *C. auris* biofilms in the spread of infection and the induction of resistance. Here, I shed light on the potential role of *C. auris* dispersed cells in resistance development. Initially, the resistance profile of *C. auris* dispersed cells compared to that of the parent planktonic cells were explored. While many reports have highlighted that the cells in biofilms are more resistant to antimicrobial agents than are the parent planktonic cells [16–18], little is known about the cells dispersed from biofilms. This prompted us to explore the resistance profile of *C. auris* dispersed cells to antifungal drugs. The antifungal drugs used in the present study were selected to represent the major available antifungal groups (azoles, polyenes, fluoro-pyrimidine, and echinocandin) [19]. With the exception of amphotericin B, no difference was found in the resistance profiles of the parent planktonic cells and the dispersed cells. To provide more insight, gene expression analysis of resistance genes was performed. Surprisingly, the dispersion of cells from biofilm resulted in greater induction of resistance genes, although similar antifungal susceptibility was observed. This suggests that dispersed cells may have a heightened potential to develop resistance over time, even though the current MIC_50_ differences are minimal. The disparity between the gene expression levels and the MIC_50_ values could indicate that while the resistance mechanisms are being activated, they may not yet have manifested fully in terms of drug susceptibility. Hence, it is crucial that additional research is required to better understand this inconsistency, including more sensitive assays or long-term studies to observe whether dispersed cells eventually develop a clinically significant resistance phenotype. The resistance genes selected for the present study were *ERG2*, *ERG6*, *ERG11*, *CHS1*, *CHS2*, *FKS1*, *CDR1*, and *MDR1*. Ahmad et al. showed that both the *ERG6* and *ERG2* genes are targets that confer reduced susceptibility to amphotericin B in *Candida glabrata* [20]. Another study highlighted that a mutation in *ERG6* is responsible for the emergence of amphotericin B resistance in *C. auris* and can cause a more than 32-fold increase in amphotericin B resistance [21]. Mutation or overexpression of the *ERG2/ERG6* gene impacts the biosynthesis of amphotericin B target (ergosterol) and consequently abrogates its bioactivity [21]. The expression of the *ERG11* gene encodes lanosterol demethylase, the target of azole antifungals; hence, mutation or overexpression of *ERG11* mitigates azole bioactivity [2]. For instance, overexpression of the *ERG11* gene increased the fluconazole MIC_50_ in *C. auris* by 3-fold [2]. Similar findings were reported for *ERG11* overexpression in *C. albicans* [22]. On the other hand, overexpression of the *CHS1*, *CHS2*, and *FKS1* genes is considered the main mechanism underlying the emergence of echinocandin resistance in *Candida* species [23, 24]. The *CHS1* and *CHS2* genes encode chitin synthase enzymes, while the *FKS1* gene encodes 1,3-β-d-glucan synthase [24, 25]. We previously observed that overexpression of the *CHS1*, *CHS2*, and *FKS1* genes elevated the caspofungin MIC_50_ more than 10-fold [2]. Similarly, Davari et al. reported similar results for *C. parapsilosis* [26]. Genes encoding membrane transport proteins of the ABC transporter (*CDR*1) and the major facilitator (*MDR1*) play a primary role in resistance emergence in *Candida* species [27–29]. Notably, overexpression of both genes can induce resistance to fluconazole and caspofungin in *Candida* species. For instance, fluconazole-resistant *C. albicans* isolated from Ahvazian cancer patients exhibited overexpression of the *CDR1*,* CDR2*, and *MDR1* genes [28]. Similar findings were reported by EL-Kholy et al. [30]. Future work should focus on comparing the gene expression of dispersed cells with biofilm-associated cells. This comparison could provide additional insights into whether the observed resistance is specifically linked to the dispersal process or biofilm adaptation.

It was reported that *C. auris* can acquire multidrug resistance following exposure to caspofungin by overexpressing the *CDR1* gene [2]. This prompted us to question whether the recorded overexpression of resistance genes, particularly the *CDR1* and *MDR1* genes, in dispersed cells can influence their ability to acquire resistance once exposed to antifungal drugs. In this context, *C. auris* dispersal cells and parental planktonic cells were exposed to caspofungin at sub-MIC levels, after which the MIC_50_ was measured. We previously showed that *C. auris* can acquire resistance to caspofungin once exposed to sub MIC level [2]. Caspofungin was selected for this experiment because it is the first-line antifungal agent for treating invasive *C. auris* infections, as recommended by CDC and other global health authorities [31]. Interestingly, *C. auris*-dispersed cells exhibited greater resistance to caspofungin than did the parental planktonic cells. This finding is of great interest because caspofungin is the first-line therapy for treating invasive *C. auris* infection [32]. Additionally, a previous report highlighted the death of C. *auris*-infected patients due to the ability of *C. auris* to acquire multidrug resistance during caspofungin therapy [33]. Moreover, to test whether dispersed cells can form distinct biofilms, I allowed planktonic and dispersed cells to form biofilms under the same conditions. Strikingly, dispersed cells formed more viable biofilms. This observation indicated that dispersed cells display a distinct phenotype from that of their parent planktonic cells. Future research should incorporate additional methods to provide a more comprehensive understanding of biofilm formation and characteristics in dispersed cells. To investigate whether the observed magnitude of biofilm viability was related to adhesion capacities or differential maturation rates, the expression of genes involved in biofilm initiation and maturation was explored. *ALS* genes are involved in the synthesis of a family of cell-surface glycoproteins in *Candida* species [34]. Cell surface glycoproteins play a crucial role in the pathogenesis of *Candida* species by mediating the adherence of *Candida* to host tissue [35–37]. The *ALS5* gene encodes for the synthesis of agglutinin-like protein 5, which plays a major role in resistance development by *C. auris* in addition to its role in biofilm formation. Notably, the *ALS5* gene induced self-aggregation of *C. auris* when exposed to caspofungin, which consequently provoked physical resistance to fluconazole and caspofungin in *C. auris* [2]. Furthermore, several reports have highlighted the role of the *ALS5* gene in the attachment of yeast cells to polystyrene surfaces and in biofilm formation [38, 39]. For instance, Kean et al., showed that the *ALS5* gene is upregulated by more than 3-fold during *C. auris* biofilm formation [39]. On the other hand, the *KRE6* gene encodes for the synthesis of β-1,6-glucan, which is a major component of *Candida* biofilms [40, 41]. Transcriptome analysis of *C. auris* biofilms revealed the potential role of the *KRE6* gene in *C. auris* biofilm maturation [39]. Although increased expression of both the *ALS5* and *KRE6* genes was observed in the biofilm formed by dispersed cells compared to parent planktonic cells, the fold changes were less than two. While these changes suggest a potential contribution of *ALS5* and *KRE6* to biofilm formation, such expression levels may not be biologically significant. Consequently, while these genes may play a role in the enhanced biofilm-viability of the dispersed cells, further studies are needed to confirm their impact. Transitioning from the genetic basis of the acquired resistance and biofilm formation of *C. auris* dispersed cells, it is equally important to examine the metabolic profile of these cells as the metabolites produced by *Candida* play a significant role in its ability to thrive in diverse environments and contribute to its virulence and resistance [42]. The different metabolic profile observed for *C. auris* dispersed cells may indicates that dispersed cells can adapt their metabolism to survive in various environments. This flexibility allows them to exploit different nutrient sources and survive under stress conditions that planktonic cells might not withstand. For instance, glyoxylate and dicarboxylate metabolism was the most significantly enriched metabolic pathway in *C. auris* dispersed cells unlike the parent planktonic cells. *Candida* can activates the glyoxylate cycle under nutrient-limited conditions, such as low glucose concentration [43, 44]. Additionally, glycerolipid metabolism was observed to be enriched at a higher ratio compared to the parent planktonic cells. Glycerolipids are crucial components of the microbial cell membrane, contributing to its integrity and fluidity, and thus can confer resistance to antifungal treatments [45, 46]. Also, yeast can switch to glycerolipid metabolism under stress condition [45]. Another notable observation was the clear activation of the TCA cycle in the *C. auris* dispersed cells. The activation of the TCA cycle leads to increased ATP levels, which are necessary for various cellular functions and stress responses [47]. High ATP levels are associated with the activation of the Ras1-cAMP signaling pathway, which promotes hyphal development and virulence​ [47]. Interestingly, the activation of the TCA cycle can enable *C. auris* dispersed cells to utilize non-fermentable carbon sources for energy production, crucial for survival and growth in environments where fermentable sugars are scarce, such as within macrophages during infection [44]. ​ Among the unique metabolites produced by *C. auris* dispersed cells, were sugar alcohols (polyols) such as arabitol, ribitol, and carbitol, in addition to the overproduction of glycerol. Yeast can tolerate high external osmotic environments by producing additional polyols [48, 49]. For instance, glycerol can function either as an osmolyte, maintaining water balance, or as an osmoprotectant, facilitating many cellular processes during osmotic stress [49, 50]. Additionally, the accumulation of polyols can enhance yeast viability when exposed to oxidative and high-temperature stress [51]. Another major unique metabolite that was solely produced from *C. auris* dispersed cells is phenylethyl alcohol. It is one of the first quorum sensing molecules identified in *Candida* [52]. Quorum-sensing molecules play a crucial role in morphogenesis, virulence, and biofilm formation [53]. It facilitates microbial communication within a population. This occurs through the continuous release of quorum-sensing molecules. As the concentration of these molecules increases in proportion to the population size, reaching a critical threshold, triggers a response. This response leads to the coordinated expression or repression of target genes related to quorum sensing [53]. Quorum-sensing molecules can additionally protect *Candida* from oxidative stress [54]. Another study highlighted the ability quorum-sensing molecule to triggers the expression of genes encoding drug efflux that confer drug resistance [55]. Further, it was found that phenylethyl alcohol is involved in nitrogen- and signalling-mediated morphogenesis in *Saccharomyces cerevisiae* under starvation condition [56]. It is beyond the argument that our data led us to believe that cells dispersed from *C. auris* biofilms can demonstrate a greater ability to recolonize surfaces and cause resistant infections, as they can adhere more easily to form biofilms. They also able to switch to other metabolic pathways to survive under stress and nutrient limited condition. From the other side, the unique metabolite profile of dispersed cells can serve as biomarkers for detecting biofilm-related infections and monitoring treatment efficacy. Further, understanding the specific metabolic pathways active in dispersed cells can lead to the development of targeted therapies that disrupt these pathways, making the cells more susceptible to existing treatments. Hence, it seems fundamental in the future to perform more studies to carefully investigate the pathogenesis of cells dispersed from *C. auris* biofilms. For instance, we need to understand their invasion capability and their ability to resist innate immune defenses compared to those of parental planktonic cells.

## Conclusion

This study demonstrated that cells dispersed from *C. auris* biofilms may not revert to the planktonic state once released from the biofilm. These dispersed cells express resistance genes more strongly than their parent planktonic cells and have acquired resistance to caspofungin. Additionally, they form more viable biofilms compared to the parent planktonic cells and exhibit an altered metabolic profile that enhances their viability under stress conditions. It is crucial that further studies are required to confirm the stability of these changes over several generations. These characteristics highlight the importance of considering biofilm dynamics and Candida’s metabolic adaptability when developing diagnostic tools and treatment strategies. By targeting the unique metabolic pathways of dispersed cells, more effective interventions for preventing and treating *Candida* infections may be developed.

## Data Availability

The metabolomic data have been uploaded to the Metabolomics Workbench, an international repository for metabolomics data and metadata. They can be accessed at http://dx.doi.org/10.21228/M8424B (Project Number: PR002232).
